# The Metabolic Inhibitor CPI-613 Negates Treatment Enrichment of Ovarian Cancer Stem Cells

**DOI:** 10.3390/cancers11111678

**Published:** 2019-10-29

**Authors:** Chiara Bellio, Celeste DiGloria, David R. Spriggs, Rosemary Foster, Whitfield B. Growdon, Bo R. Rueda

**Affiliations:** 1 Vincent Center for Reproductive Biology, Department of Obstetrics and Gynecology, Massachusetts General Hospital, Boston, MA 02114, USA; chiarabellio87@gmail.com (C.B.); CDIGLORIA@mgh.harvard.edu (C.D.); RFOSTER1@mgh.harvard.edu (R.F.); WGROWDON@MGH.HARVARD.EDU (W.B.G.); 2 Obstetrics, Gynecology and Reproductive Biology, Harvard Medical School, Boston, MA 02115, USA; 3 Department of Medicine, Massachusetts General Hospital, Boston, MA 02114, USA; Dspriggs@mgh.harvard.edu; 4 Department of Medicine, Harvard Medical School, Boston, MA 02115, USA; 5 Division of Gynecologic Oncology, Department of Obstetrics and Gynecology, Massachusetts General Hospital, Boston, MA 02114, USA

**Keywords:** cancer stem cells (CSCs), ovarian cancer chemoresistance, metabolic inhibitor, combinational therapy

## Abstract

One of the most significant therapeutic challenges in the treatment of ovarian cancer is the development of recurrent platinum-resistant disease. Cancer stem cells (CSCs) are postulated to contribute to recurrent and platinum-resistant ovarian cancer (OvCa). Drugs that selectively target CSCs may augment the standard of care cytotoxics and have the potential to prevent and/or delay recurrence. Increased reliance on metabolic pathway modulation in CSCs relative to non-CSCs offers a possible therapeutic opportunity. We demonstrate that treatment with the metabolic inhibitor CPI-613 (devimistat, an inhibitor of tricarboxylic acid (TCA) cycle) in vitro decreases CD133+ and CD117+ cell frequency relative to untreated OvCa cells, with negligible impact on non-CSC cell viability. Additionally, sphere-forming capacity and tumorigenicity in vivo are reduced in the CPI-613 treated cells. Collectively, these results suggest that treatment with CPI-613 negatively impacts the ovarian CSC population. Furthermore, CPI-613 impeded the unintended enrichment of CSC following olaparib or carboplatin/paclitaxel treatment. Collectively, our results suggest that CPI-613 preferentially targets ovarian CSCs and could be a candidate to augment current treatment strategies to extend either progression-free or overall survival of OvCa.

## 1. Introduction

Ovarian cancer (OvCa) is the most lethal gynecologic malignancy in the United States. It is estimated that approximately 22,530 women will be newly diagnosed in 2019, while it is expected that roughly 13,980 women will succumb to the disease [1]. Ovarian cancer is classified by different subtypes based on histological features [2], with the majority being of an epithelial origin. High grade serous ovarian cancer (HGSOC) is the most common of ovarian epithelial cancers [3]. The most challenging aspect of treating HGSOC is that most patients present with advanced stage. The lack of an accurate detection method for early-stage disease contributes to poor survival rates [4]. Current treatment paradigms for advanced HGSOC include cytoreductive surgery followed by platinum- and taxane-based chemotherapy or upfront neoadjuvant chemotherapy, followed by interim cytoreductive surgery [5]. Although most patients treated with first-line chemotherapy achieve a clinical response, the rate of disease recurrence remains high [6]. Unfortunately, the survival rate of recurrent OvCa is poor since recurrent disease is often more resistant to traditional treatment strategies [7]. Many anticancer agents, including poly-ADP-ribose polymerase (PARP) inhibitors and combination therapy, have been developed and are being validated for use in the recurrent HGSOC setting, though overall survival benefits are unclear [8].

Mounting research suggests that OvCa stem cells (CSCs) contribute to recurrent and platinum-resistant disease [9,10,11]. The CSC hypothesis suggests tumors are hierarchically organized with a subpopulation of cancer cells at the top with self-replicating properties [12,13]. Furthermore, it is postulated this relatively rare population within the bulk tumor retains or acquires other stem-like features including, the ability to give rise to more differentiated daughter cells, infrequent turnover, drug resistance, and inherent and/or acquired capacity to repair DNA [14,15]. All these properties can contribute to that pathology, maintenance, or recurrence of the disease [16,17,18]. The CSC theory has recently incorporated the concept of CSC plasticity, which proposes that there are sub-sets of cells that, when under stress, have the bidirectional capacity to dedifferentiate or differentiate displaying a range of stem-like properties [19,20,21,22]. We hypothesize that the elimination or suppression of CSCs is a critical step in the development of effective treatment strategies that prevent OvCa cell resurgence and lead to a more durable clinical response. Furthermore, a more in-depth understanding of the molecular features associated with the CSC phenotype is vital to develop effective treatments [23].

It is well-accepted tumor cells gain a survival benefit by relying on differential tumor metabolism [24,25]. More specifically, cancer cells, as opposed to normal cells, preferentially metabolize glucose by glycolysis instead of oxidative phosphorylation, even in the presence of oxygen, mechanism well known as the “Warburg effect” [26]. The accelerated glycolysis adaptation in tumors is believed to contribute, in part, to their chemoresistance [27,28]. Moreover, this upregulated metabolism generates more lactate via lactate dehydrogenase, causing an acidification of the tumor microenvironment that is known to facilitate tumor invasion and metastasis [29,30]. Consequently, combination therapeutic strategies utilizing classical cytotoxic drugs together with agents designed to disrupt cellular metabolism in tumors cells have been developed [31,32,33]. While these combination strategies may prove to be effective, the durability of their response remains questionable. This uncertainty is due in part to the increasing evidence which supports the concept that unlike normal stem cells, which use oxidative phosphorylation as their primary source of energy, or cancer cells, which are highly glycolytic, CSCs demonstrate unique metabolic flexibility [34]. This concept, on the surface seems controversial, due to different to studies demonstrating that CSCs are either mainly glycolytic or suggesting mitochondrial metabolism as a principal source of energy [35,36]. However, it is more likely that CSCs can adapt their metabolism by shifting energy production from one pathway to another as conditions within the tumor microenvironment change [37]. Several agents targeting many different pathway components contributing to general cancer cell metabolism are currently approved or are being tested in clinical trials [38], but only a small number of studies suggest specific metabolic inhibitor targeting CSCs [39,40]. CPI-613, (devimistat, an inhibitor of tricarboxylic acid (TCA) cycle) is a member of the anti-cancer lipoate derivatives that target specifically two enzymes involved in the mitochondrial tricarboxylic acid (TCA) cycle: pyruvate dehydrogenase (PDH) and alpha-ketoglutarate dehydrogenase (KGDH) [41,42]. The Food and Drug Administration (FDA) designated CPI-613 as an orphan drug for the treatment of acute myeloid leukemia (AML), pancreatic cancer, myelodysplastic syndromes (MDS), and more for the treatment of Burkitt’s lymphoma and peripheral T-cell lymphoma (PTCL). In clinical trials, CPI-613 has been shown to be specific for targeting tumor cells with a low systemic toxicity profile in patients [43,44]. Despite these promising results in other malignancies, there is no evidence that CPI-613 would be effective in the treatment of OvCa. Recent studies suggest mitochondrial metabolic heterogeneity in OvCa tumor samples could be utilized to monitor OvCa development and progression after surgery and therapy [45]. Dar and colleagues demonstrated that OvCa cells display heterogeneity in their use of glycolysis or oxidative phosphorylation. These authors postulated that the flexibility in using different metabolic pathways as a means of survival in response to environmental changes or drug-selective pressure suggested was a self-regulated desire for “cellular fitness” and associated with OvCa chemoresistance [46]. Other studies also investigated the metabolic phenotype of ovarian CSCs to support the idea that they display metabolic plasticity and heterogeneity [47,48]. In 2014, Pastò and colleagues provided support for the conclusion that cluster of differentiation (CD)44+CD117+ OvCa cells preferentially utilize oxidative phosphorylation and resist glucose deprivation. Specifically, they showed that ovarian CSCs are characterized by a higher level of mitochondrial reactive oxygen species (ROS) production and elevated mitochondrial membrane potential compared to their non-CSC counterparts [49]. This finding is important for the development of alternative treatment modalities since we and others have shown that carboplatin/paclitaxel, or the more innovative PARP inhibitors, as olaparib, result in an enrichment of ovarian CSCs which likely contribute to chemoresistance and recurrence [11,50]. Disrupting the CSC source of energy may be critical to their demise.

We hypothesized that CPI-613, a mitochondrial metabolic inhibitor, could preferentially target ovarian CSCs and augment the impact of the more standard cytotoxic therapies that often leave CSCs behind. This investigation provides evidence that the metabolic inhibitor CPI-613 preferentially targets ovarian CSCs. Moreover, a combination of CPI-613 and carboplatin/paclitaxel or olaparib complement each other as evidenced by a reduction in the carboplatin/paclitaxel and PARPi induced increase in CSC frequency in our in vitro and in vivo models of OvCa.

## 2. Results

### 2.1. CD133 and CD117 Marker Expression in OvCa Cell Lines Identifies a Cell Population with Stem-Like Features

Many studies have utilized CD133 and CD117 surface antigens as markers of ovarian CSCs [51]. Moreover, we and others have contributed to the abundance of reports demonstrating the pro-tumorigenic potential of the CD133 and CD117 positive tumor cell fractions relative to the non CD133 and CD117 enriched cell fractions [11,52,53]. Before testing the impact of CPI-613 on a subpopulation of ovarian CSCs, the baseline CD133 and CD117 levels were determined in the five ovarian serous cancer cell lines used in our study. All analyses were initiated by gating on viable cells and then determining the percent cells that expressed CD133 and CD117. As expected, the levels were variable among the five different cell lines (Table S1).

CSCs are typically resistant to chemotherapy, in part due to the expression of drug-extruding pumps and detoxifying enzymes [18]. This observation was confirmed by finding the percentage of CD133+ and CD117+ cells increased dramatically following in vitro treatment of OvCa cell lines with carboplatin (Figure S1A). The increase in CD133+ and CD117+ cells corresponded with a decrease in CD133− and CD117− cells. Another key feature of CSCs is their capacity for self-renewal. To define the functional stem cell properties of these cell lines, we performed the sphere assay in all the cell lines. CSCs usually display a survival advantage in sphere-forming conditions, such as low adhesion plates and in the absence of serum. Spheroid formation was associated with a decrease in total cell viability. However, there was an enrichment of CD133+ and CD117+ cells frequency (Figure S1B), further supporting their stem-like potential. Together, these results indicate that the CD133+ and CD117+ cells in all the cell samples analyzed display some CSC-like properties.

### 2.2. CPI-613 In Vitro Induces a Decrease in CD133 and CD117 Positive Cell Frequency

**Figure 1:**
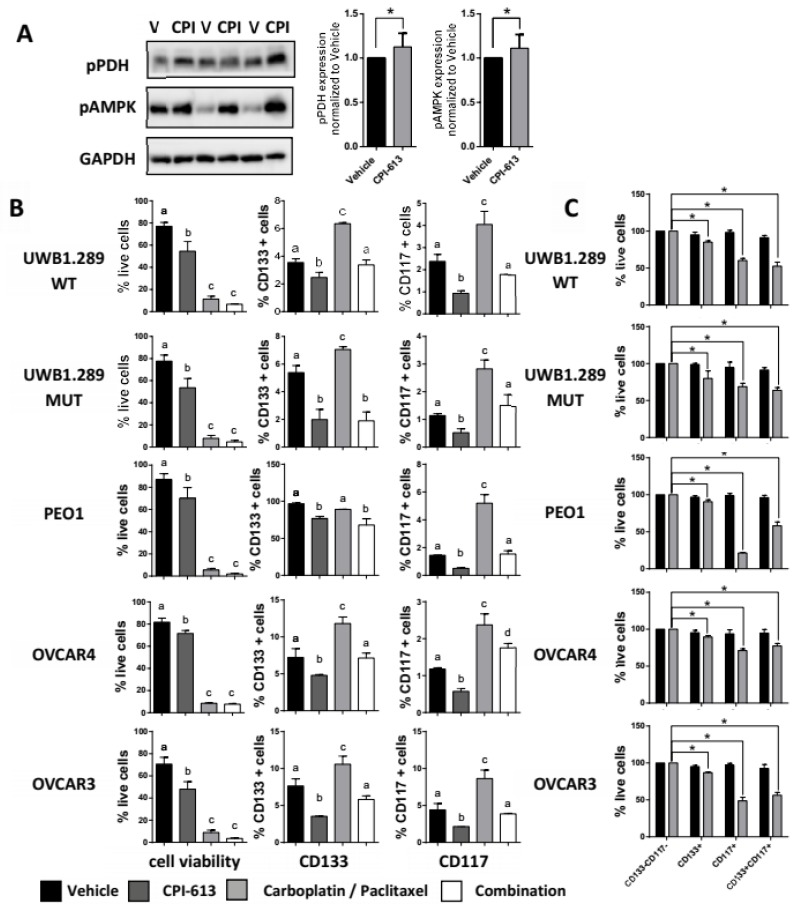

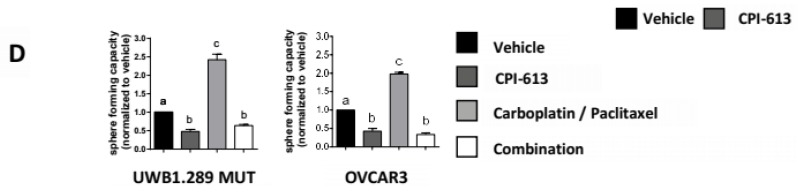
CPI-613 in vitro treatment induces a decrease in CD133+ and CD117+ cells frequency and sphere-forming capacity. (**A**) Western blot analysis of phospho-pyruvate dehydrogenase (pPDH) and phospho-AMP-activated protein kinase (pAMPK) levels in cultured cells treated every 72 h with 75 µM CPI-613 for a 7 d period to confirm on target effects of CPI-613. Protein levels were quantified from three independent experiments. Normalization was done with glyceraldehyde 3 phosphate dehydrogenase (GAPDH), as a loading control. * *p*-value < 0.05. (**B**) Flow cytometric determination of the frequency of viable (left panels), CD133+ (center panels) and CD117+ (right panels) cells in the indicated cell lines following treatment (every 72 h for a 7 d period) with 75 µM CPI-613, 5 µM (UWB1.289 MUT and UWB1.289 WT) or 10 µM (PEO1, OVCAR4 and OVCAR3) carboplatin/2 nM (UWB1.289 MUT and UWB1.289 WT) or 1 nM (PEO1, OVCAR4, and OVCAR3) paclitaxel and combination in vitro. The graph for each cell line represents the mean percentage ± SEM calculated from three independent experiments. Different letters indicate statistically significant differences between groups, *p*-value < 0.001. (**C**) Relative cell viability of CD133−CD117−, CD133+, CD117+, and CD133+CD117+ cells as determined by flow cytometry following treatment with 75 µM CPI-613 every 72 h for a 7 d period. For each cell line, the graph shows the mean viable cell number ± SEM measured in three independent experiments. * *p*-value < 0.05. (**D**) Extreme limiting dilution analysis (ELDA) of the indicated cell lines following treatment with 75 µM CPI-613, 5 µM (UWB1.289 MUT and UWB1.289 WT) or 10 µM (PEO1, OVCAR4, and OVCAR3) carboplatin/2 nM (UWB1.289 MUT and UWB1.289 WT) or 1 nM (PEO1, OVCAR4, and OVCAR3) paclitaxel and the combination treatment in vitro every 72 h for a 7 d period. The data are expressed as the sphere-forming frequency of cells exposed to the different drugs normalized to the corresponding vehicle control and suggest the relative frequency of cells forming spheres in each sample. Three separate iterations of the analysis were performed with each cell line, and the numbers represent the mean sphere-forming frequency ± SEM. Different letters indicate statistically significant differences between groups, *p*-value < 0.001.

Mounting evidence supports the concept that CSCs display metabolic plasticity and heterogeneity relative to their non-CSC counterparts [54]. This concept was recently substantiated in an OvCa model, whereby CD44+CD117+ cancer cells were shown to prefer the mitochondrial oxidative phosphorylation metabolism instead of the high glucose uptake and lactate production despite the presence of sufficient oxygen, better known as the Warburg effect [49]. Consequently, we hypothesized that CPI-613, a metabolic inhibitor of the mitochondrial TCA cycle, would preferentially target stem-like cancer cells. To test this hypothesis, we utilized five independent platinum-sensitive and *TP53* mutated HGSOC lines: UWB1.289 WT, UWB1.289 MUT, PEO1, OVCAR4, and OVCAR3 (Figure S1A). The cells were treated with CPI-613 and CD133/CD117 levels, and cell viability were measured 7 days following the treatment by flow cytometry. Initially, we conducted dose-response curves with CPI-613 with all cell lines. Based on our results, we selected 75 µM as our treatment concentration, given that it was near the IC50 (half maximal inhibitory concentration) for all cell lines. Carboplatin/paclitaxel treatment was used as a positive control since it has been demonstrated to increase the frequency of CSCs [55]. Initially, we tested the effect of CPI-613 on its target enzyme pyruvate dehydrogenase (PDH) by assessing the expression of the phosphorylated form of PDH (pPDH), the inactivated form of the protein [56]. To determine whether CPI-613 directly inhibited mitochondrial metabolism, we used the UWB1.289 MUT cell line. The cells were harvested following acute exposure (2 h) of CPI-613, and the lysates were subjected to a Western blot with an antibody that is specific for the phospho S293 residue of the PDH E1 subunit of the protein. Treatment with CPI-613 induced an increase in pPDH, demonstrating that a short time window was sufficient to observe the inhibitory effect of the drug (Figure 1A and Figure S3). To confirm that CPI-613 targets the TCA mitochondrial metabolic cycle by depleting cellular adenosine triphosphate (ATP), we assessed the phosphorylation status of 5’ adenosine monophosphate-activated protein kinase (AMPK). AMPK is phosphorylated in the presence of a high adenosine monophosphate (AMP)/adenosine diphosphate (ADP) ratio as a consequence of ATP depletion in the cells [45]. Acute treatment with CPI-613 resulted in an increase in AMPK phosphorylation (Figure 1A and Figure S3). These data confirmed that CPI-613 targets mitochondrial metabolism in our model. CPI-613 treatment alone was associated with a decrease in CD133+ and CD117+ cell frequency in all the cell lines compared to carboplatin/paclitaxel treatment which either had no (PEO1) or induced the expected CSC enrichment (Figure 1B). While carboplatin/paclitaxel alone was more efficient than CPI-613 in reducing overall tumor cell viability, its lack of negative effects on the CSC populations was clear (Figure 1B, left panel) again suggesting a preferential effect of CPI-613 on the CSCs. The combination of CPI-613 and carboplatin/paclitaxel had the capacity to offset either the resistance or enrichment of CD133+ and CD117+ cell frequency observed in response to the carboplatin/paclitaxel treatment alone (Figure 1B, *p*-value < 0.001). To demonstrate that CPI-613 specifically targeted the CD133+ and CD117+ cells, we performed post-treatment flow-cytometry analysis to determine the cell viability of CD133−CD117−, CD133+, CD117+, and CD133+CD117+ cell fractions (Figure 1C). The graphs reveal that CPI-613 treatment at 75 µM had a negative effect on the survival effect of CD133+, CD117+, and CD113+CD117+ cells, with a more significant decrease in the CD117+ fraction. In contrast, CD133−CD117− cell population did not show any change in viability, confirming the CPI-613 target effect was primarily on CSCs. Finally, to corroborate these data, we confirmed the CSC properties after CPI-613 treatment analyzing the sphere-formation efficiency in a limiting-dilution assay (Figure 1D). UWB1.289 MUT and OVCAR3 cells that survived the CPI-613 treatment demonstrated a lower rate of sphere-forming capacity compared to vehicle and the enriched cells after carboplatin/paclitaxel treatment. Combinational treatment between CPI-613 and carboplatin/paclitaxel showed a similar effect on sphere formation efficiency to CPI-613 single agent, confirming the overwhelming effect on the CSC enrichment after carboplatin/paclitaxel treatment.

### 2.3. CPI-613 Treatment Negatively Impacts CSC-Rich Spheres and Results in a Decrease in Tumorigenicity In Vivo

**Figure 2:**
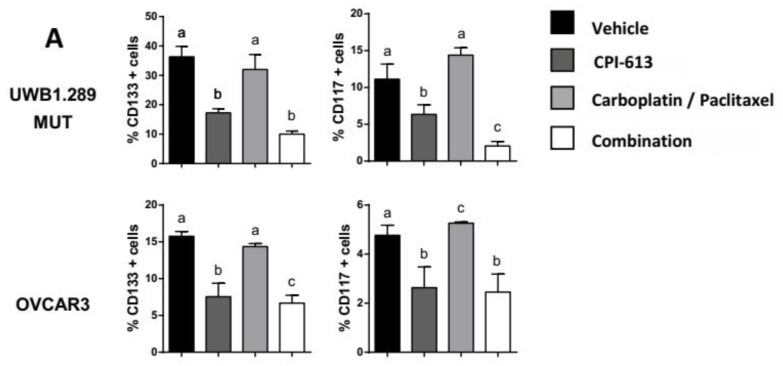

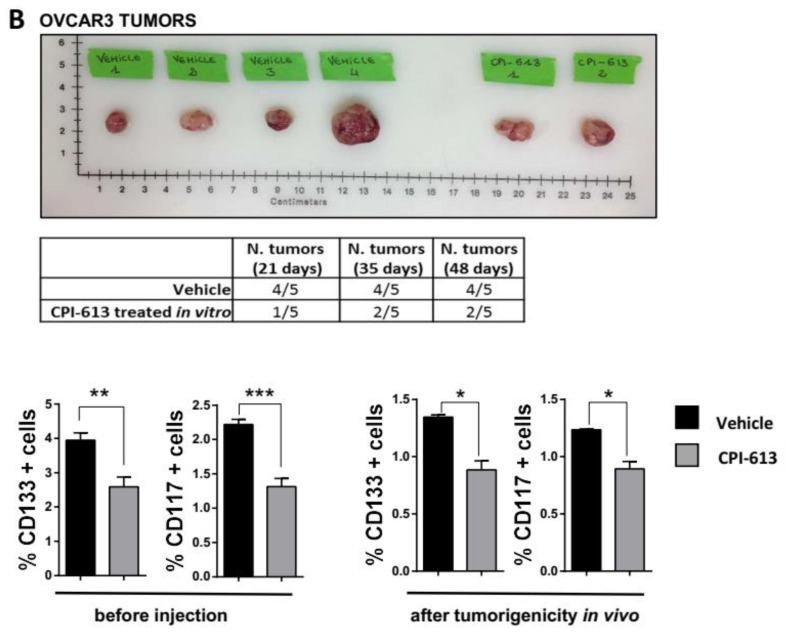
CPI-613 in vitro treatment induces a decrease in CD133+ and CD117+ cells in CSC-rich spheres and affects tumorigenicity capacity in vivo. (**A**) Flow cytometric determination of the frequency of CD133+ (left panels) and CD117+ (right panels) cells in UWB1.289 MUT and OVCAR3 spheres following treatment with 75 µM CPI-613, 5 µM (UWB1.289 MUT and UWB1.289 WT) or 10 µM (PEO1, OVCAR4, and OVCAR3) carboplatin/2 nM (UWB1.289 MUT and UWB1.289 WT) or 1 nM (PEO1, OVCAR4, and OVCAR3) paclitaxel and combination. Treatments were administered every 72 h for 7 d. The graph for each cell line shows the mean percentage of frequency ± SEM calculated from three independent experiments. Statistical comparison between vehicle and combination treatment in each cell line. Letters (a, b, c, d) indicate the multiple comparison results. The same letters indicate no significant difference; different letters indicate significant differences, *p*-value < 0.001. (**B**) The table shows the tumorigenicity rate of cells pretreated in vitro with vehicle and 75 µM CPI-613 every 72 h for 7 d. Representative figure of tumors sizes after harvest at day 48 (upper panel) and flow cytometry analysis of CD133 and CD117 expression frequency before injection of cells and after 48 d of tumor formation (lower panel). SEM derived by three different tumors and statistical comparison between vehicle and CPI-613 pretreated in vitro arms, *p*-value < 0.01. * *p*-value < 0.05; ** *p*-value < 0.01; *** *p*-value < 0.001. One of the most important properties of CSCs is their enhanced ability to form tumors in vivo when compared with non-CSCs [58]. To provide additional evidence that CPI-613 preferentially targets CSCs, we pretreated OVCAR3 cells in vitro with 75 µM CPI-613 or vehicle for 7 d and then injected 1 × 10^6^ cells per mouse. The onset of tumor formation was checked weekly. Given that the time needed for evidence of OVCAR3 tumor formation at the cell concentration of 1 × 10^6^ cells is approximately three weeks post-injection (21-d checkpoint on the table of Figure 2B), the mice were euthanized after 48 d and tumors were harvested. CPI-613 pretreated cells demonstrated a delay in forming tumors compared to vehicle pretreated cells. Specifically, only one mouse formed a tumor after 21 d compared to the four mice from the vehicle pretreated arm (Table of Figure 2B). To assess whether the CPI-613 induced reduction of CD133+ and CD117+ cells was maintained during the latency time, we assessed the CD133+ and CD117+ cell frequency after pretreatment in vitro and again when the tumors were harvested 48 d post-inoculation with tumor cells. The flow-cytometry analysis revealed the CD133+ and CD117+ cell frequency in the tumors was less in cells pretreated with CPI-613 relative to those pretreated with vehicle (Figure 2B), suggesting the effect of CPI-613 treatment on the CSC population might be sustained for some time.

Many studies provide evidence that sphere-forming conditions enrich CSCs in vitro [57]. To confirm the CPI-613 target effect on the CSC population, we treated UWB1.289 MUT and OVCAR3 spheres after incubating these cells for 14 days in sphere promoting culture conditions in low-adhesion plates (Figure 2A). In contrast to what was observed in the previous monolayer experiments [50], carboplatin/paclitaxel treatment, used as positive control, had no effect (*p*-value > 0.05) on CD133+ and CD117+ cell frequency at this concentration of drug under these sphere-forming culture conditions which preferentially enrich for CSCs when compared to vehicle, indirectly confirming CSC resistance to cytotoxics. Interestingly, CPI-613 treatment decreased CD133+ and CD117+ cell frequency (*p*-value < 0.01) in CSC-rich spheres, corroborating its target effect on CSC population. Combining CPI-613 and carboplatin/paclitaxel on CSC-rich spheres resulted in a decrease in CD133+ and CD117+ cells frequency compared to treatment with vehicle or carboplatin/paclitaxel treatments alone (Figure 2A, *p*-value < 0.001). The only additive effect of combining CPI-613 with carboplatin/paclitaxel was observed in the CD117 population in the UWB1.289 cells.

### 2.4. CPI-613 Treatment In Vivo Induces a Decrease in CD133+ and CD117+ Cell Frequency

**Figure 3:**
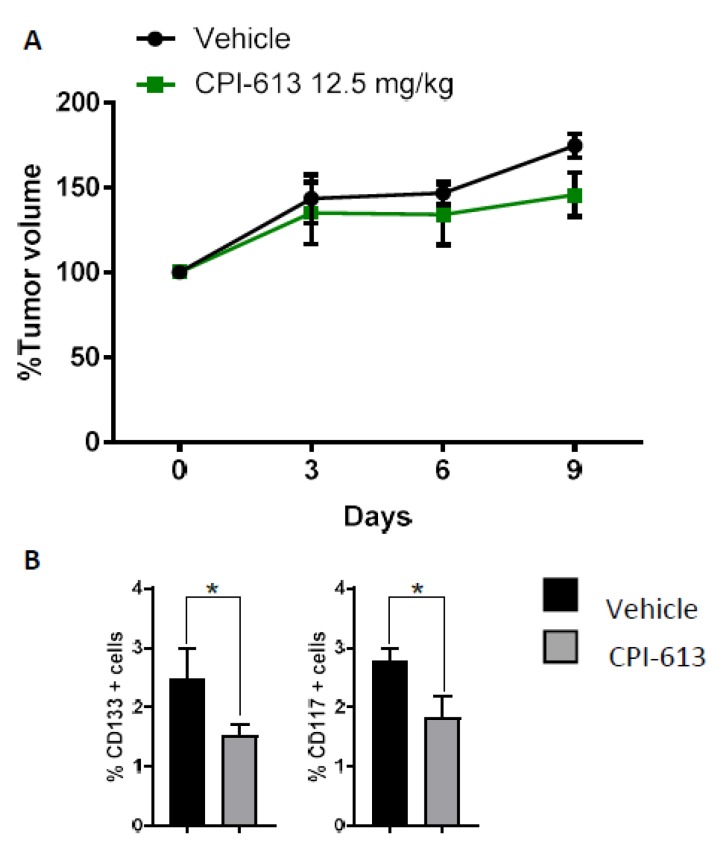
CPI-613 in vivo 10 d treatment impacts tumor growth and induces a decrease in CD133+ and CD117+ cell frequency. (**A**) Mice harboring OVCAR3-derived xenografts were randomly divided into two treatment groups. Each cohort received either vehicle or CPI-613 at the dose and administration schedule outlined in Material and Methods. The effect of treatment on tumor volume was assessed every three days. (**B**) Tumors were harvested at the end of the treatment period and stained with Annexin/PI to assess cell viability as well as CD133 and CD117 antibodies to determine the effect of CPI-613 on CD133+ and CD117+ cell frequency. In both experiments, a significant decrease (*p*-value < 0.01) in the frequency of both sub-fractions was observed following CPI-613 treatment in vivo. Each bar represents mean ± SEM. * *p*-value < 0.05.

The CPI-613 target effect on the CSC population in vitro led us to investigate whether we would observe a similar effect in vivo. OVCAR3 cells were injected s.c. in NOD/SCID (NOD.CB17-*Prkdc*^scid^/NCrCrl congenic immunodeficient) mice, and once tumors reached 200 mm^3^ of volume, the mice were treated weekly i.p. with CPI-613 at a concentration of 12.5 mg/kg. The mice were euthanized 48 h after the second injection, which was day 9 post initiating treatment (Figure 3A). At this time point, flow cytometric analysis of tumor cells revealed a decrease in CD133+ and CD117+ tumor cell frequency in CPI-613-treated mice compared with vehicle-treated mice (*p*-value < 0.001) (Figure 3B lower panel). This effect on CD133+ and CD117+ cell populations was similar to what was observed in the in vitro analysis, confirming the ability of CPI-613 to reduce CSC frequency in the tumor.

### 2.5. Combining CPI-613 and Carboplatin/Paclitaxel Treatment Impacts Tumor Growth Compared to CPI-613 Single Agent

**Figure 4:**
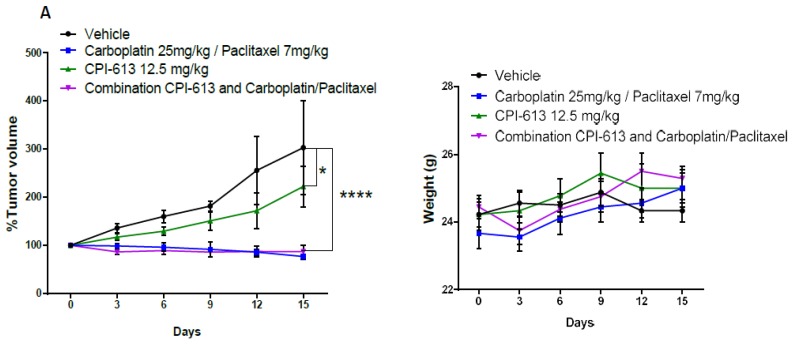

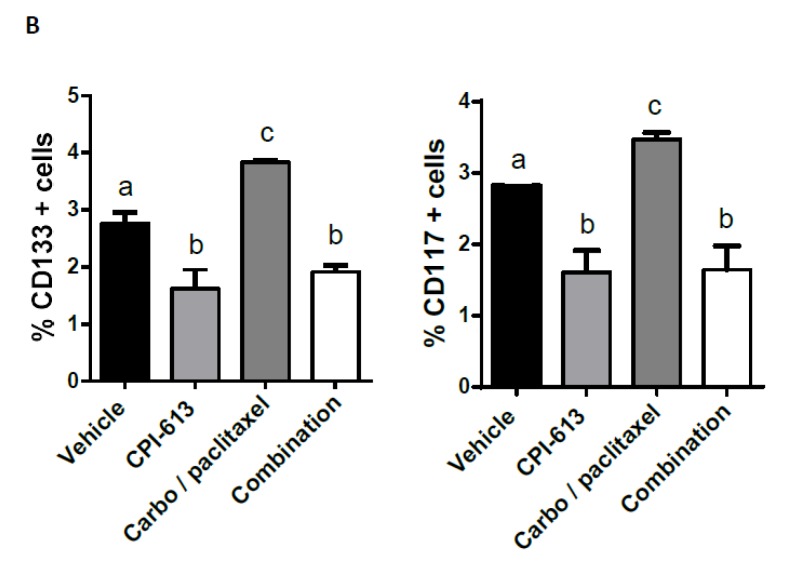
Combinational in vivo treatment of CPI-613 and carboplatin/paclitaxel impacts tumor growth compared to CPI-613 single agent. (**A**) Mice harboring OVCAR3-derived xenografts were randomly divided into four treatment groups: Vehicle, CPI-613, carboplatin/paclitaxel, and the combination of the two treatment regimens. Each cohort received drugs at the dose and administration schedule outlined in Material and Methods. The effect of each treatment on tumor volume and mouse weight was assessed every three days. Significant inhibition of tumor growth was observed in animals treated with a combination of CPI-613 and carboplatin/paclitaxel (*p*-value < 0.0001). The error bars represent SEM. * *p*-value < 0.05; **** *p*-value < 0.0001. (**B**) Tumors were harvested at the end of the treatment period, and tumor cells were stained with CD133 and CD117 antibodies to determine the effect of the different treatments on CD133+ and CD117+ cell frequency. In both experiments, a significant (*p*-value < 0.01) decrease in the frequency of both sub-fractions was observed following treatment in vivo. Different letters indicate statistically significant differences between groups (mean ± SEM, *p*-value < 0.01).

Combination drug therapy has played a particularly prominent role in the treatment of cancers as it targets multiple cancer cell-survival, promoting pathways delaying the onset of treatment resistance [59]. We demonstrated the combination of CPI-613 and carboplatin/paclitaxel in vitro negatively impacted the enrichment of chemoresistant cells in culture (Figure 1B). To assess CPI-613 in combination with carboplatin/paclitaxel in vivo, we injected OVCAR3 cells in NOD/SCID mice to compare antitumor activity of 12.5 mg/kg CPI-613 delivered once weekly either alone or in combination with carboplatin/paclitaxel (respectively 25 mg/kg and 7 mg/kg i.p. once per week). Tumor volume was assessed every 3 days. Though we used a lower dose of CPI-613 compared to other in vivo experimental protocols [60], CPI-613 single-agent inhibition of tumor growth was evident when compared to the vehicle-treated arm (*p*-value < 0.01). As expected, the carboplatin/paclitaxel treatment group and the carboplatin/paclitaxel CPI-613 combination treatment group showed reduced tumor burden as compared to the CPI-613 single agent and vehicle groups (*p*-value < 0.001; Figure 4A). More importantly, flow cytometric analysis of tumors harvested at the end of the treatment period revealed a decreased frequency of CD133+ and CD117+ cells in CPI-613 treated tumors compared with vehicle-treated controls, again implying CPI-613 preferentially targets the CSCs (*p*-value < 0.01). Of interest, however, was the combination treatment effect on CSC frequency. Despite there being no significant difference in CD133+ and CD117+ cells compared to CPI-613 single-agent treatment, the combination of CPI-613 and carboplatin/paclitaxel negated the carboplatin/paclitaxel-induced enrichment of CD133+ and CD117+ cell frequency (*p*-value < 0.001) (Figure 4B). Annexin/PI analysis of cells in the harvested tumors confirmed the increase in necrosis in the combination treatment group (Figure S2), suggesting an additive benefit of using CPI-613 in combination with classical cytotoxic.

### 2.6. Pretreatment with CPI-613 Negates the Olaparib Induced an Increase in CD133 and CD117 Positive Cells

**Figure 5:**
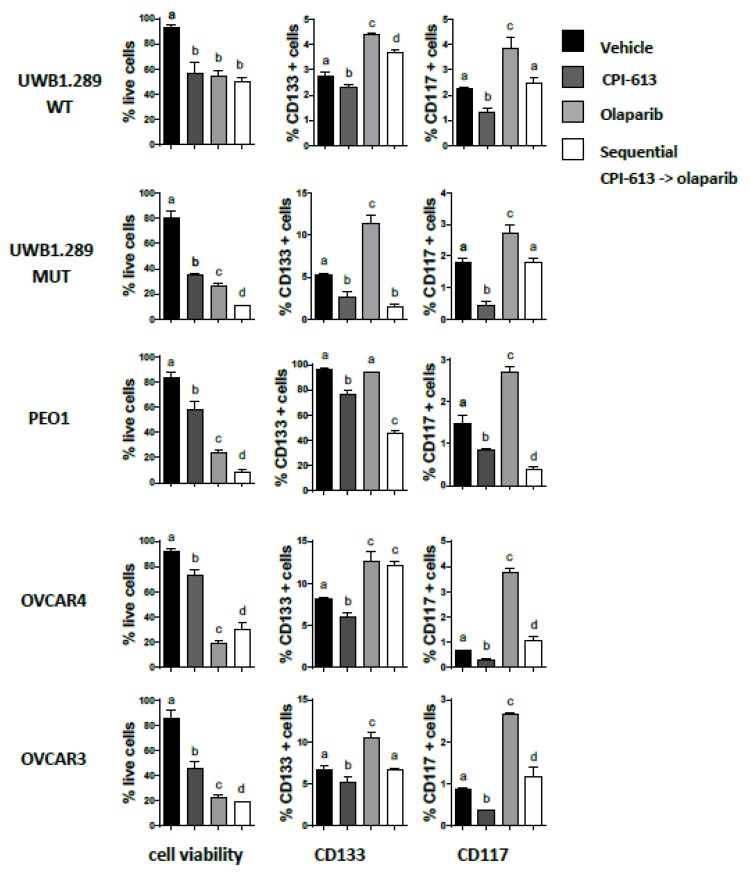
Pretreatment with CPI-613 negates the olaparib-induced increase in CD133 and CD117 OvCa positive cells. Flow cytometric determination of the frequency of viable (left panels), CD133+ (center panels) and CD117+ (right panels) cells in the indicated cell lines following treatment every 72 h with 75 µM CPI-613, 10 µM olaparib and the sequential combination in vitro for 7 d. The graph for each cell line shows the mean ± SEM calculated from three independent experiments. Different letters indicate statistically significant differences between groups, *p*-value < 0.05.

Recently we demonstrated in both in vitro and in vivo models that treatment with PARP inhibitors was less effective on ovarian CSC populations, resulting in an increase in CD133+ and CD117+ cell frequency. Presumably, these residual cells could contribute to PARP inhibitor-resistant disease and may worsen overall survival in patients receiving PARP therapy [50]. These results led us to investigate whether CPI-613 pretreatment could negate the olaparib induced increase in CSC frequency. To test this hypothesis, five OvCa cell lines were pretreated with a 75µM CPI-613 single agent for 1 week, followed by olaparib treatment for another week. Cells were harvested at the end of the sequential treatment, and the frequency of CD133 and CD117 positive cells was analyzed by flow cytometry. Except for the OVCAR4 cell line, which typically displays more resistance to treatment compared to the other cell lines in our hands, the sequential strategy induced a significant decrease in cell viability, confirming the synergy of the two drugs in the bulk population (Figure 5, left panel). Interestingly, the pretreatment with CPI-613, which preferentially targets the CSC population, decreased CD133+ and CD117+ cell frequency in all cell lines negating the enrichment confirmed by olaparib single agent (Figure 5, *p*-value < 0.001). These data suggest that pretreatment with CPI-613 negatively impacts CSCs and overcomes the unintended effect of olaparib on CSC function. Sequential treatment may, therefore, prevent or delay the anticipated tumor recurrence.

### 2.7. The Combination of CPI-613 and Olaparib Impacts Tumor Growth and Olaparib Induced Enrichment of CD133+ and CD117+ Cells

**Figure 6:**
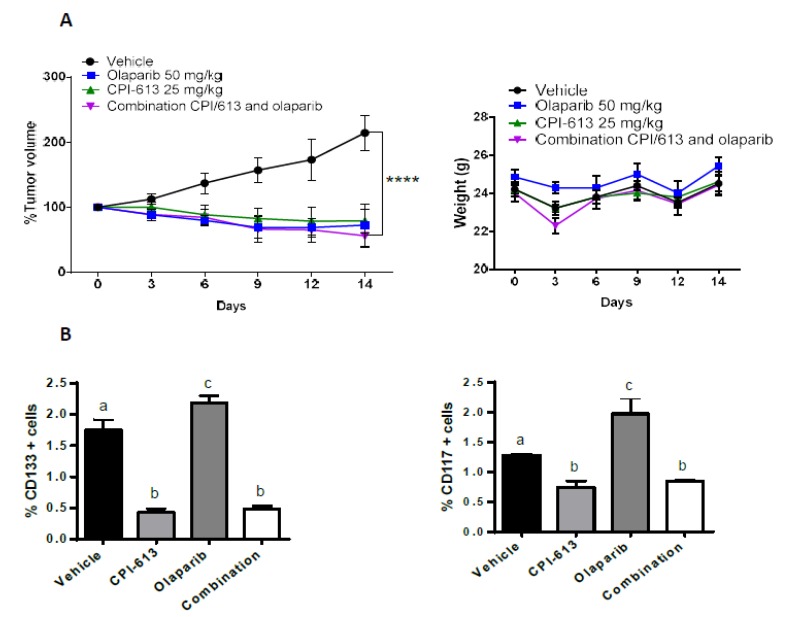
Combinational in vivo treatment of CPI-613 and olaparib impacts tumor growth and olaparib induced enrichment of CD133+ and CD117+ cells. (**A**) Mice harboring OVCAR3-derived xenografts were randomly divided into four treatment groups: Vehicle, CPI-613, olaparib, and combination. Each cohort received drugs at the dose and administration schedule outlined in Material and Methods. The effect of treatment on tumor volume and mouse weight was assessed every three days. Inhibition of tumor growth was observed in animals treated with a combination of CPI-613 and olaparib (*p* < 0.0001). The error bars represent mean ± SEM. **** *p*-value < 0.0001 (**B**) Tumors were harvested at the end of the treatment period and stained with CD133 and CD117 antibodies to determine the effect of the different treatments on CD133+ and CD117+ cell frequency. In both experiments, a statistically significant (*p*-value< 0.01) decrease in the frequency of both sub-fractions was observed following combinational treatment in vivo. Different letters indicate statistically significant differences between groups (mean ± SEM, *p*-value < 0.01).

The benefits of sequential treatment vs. combination treatment strategies are frequently debated. Drug combinations are often more effective but can also be more toxic than the sequential administration of monotherapies. Combination treatments can be preferable to sequential therapy for patients requiring an urgent reduction in their tumor burden [61,62]. Given these assumptions, we wanted to test the efficacy in vivo of CPI-613 and olaparib combination strategy. OVCAR3 cells were injected s.c. in NOD/SCID mice, and a 2-week course of daily olaparib treatment was performed in combination with weekly administration of CPI-613. In this experiment, CPI-613 was used at a concentration of 25 mg/kg, as in other experimental in vivo CPI-613 protocol. Olaparib and CPI-613 alone blunted tumor growth compared with vehicle control (Figure 6A). The impact was more dramatic than what was observed in the previous experiment, whereby CPI-613 was used at a concentration of 12.5 mg/kg (Figure 4A). At the end of the treatment period, it was noted that tumors harvested from mice in the single-agent olaparib treatment arm demonstrated an increase in the percentage of CD133 and CD117 positive cells compared to the tumors derived from the vehicle-treated arm. The combination of olaparib and CPI-613 negated the olaparib-induced increase (*p*-value < 0.0001; Figure 6B). These findings support the concept that the metabolic inhibitor CPI-613 could add benefit by reducing the PARPi induced CSC enhancement.

## 3. Discussion

Recurrent platinum-resistant HGSOC continues to be a therapeutic challenge, which is rarely overcome, leading to significant mortality. The current treatment options, while initially beneficial, eventually expose the more aggressive clonal cell populations displaying varying resistant phenotypes. Significant efforts have been made to interrogate molecular drivers and functionally characterize targetable weaknesses in these chemoresistant populations. Many agree that these clonal populations are attributed in part to CSCs and their inherent ability to evade the deleterious effects of modern treatment strategies and provide seed cells for the repopulation of the tumor. Whether this occurs by enhanced self-replication or induction of bidirectional plasticity promoting dedifferentiation of tumor cells may vary from tumor to tumor. Regardless, CSCs are postulated to be adaptable to different energy acquisition strategies to promote survival, and targeting their energy source could render them more susceptible to the more standard treatment strategies. We provide evidence that selective inhibition of the TCA cycle with the metabolic inhibitor CPI-613, decreases CD133+ and CD117+ cell frequency relative to vehicle treated OvCa cells, with negligible impact on non-CSC cell viability in vitro. Additionally, treatment with the metabolic inhibitor reduced CSC associated properties, including sphere forming capacity and tumorigenicity. More importantly, however, CPI-613 negated the CSCs related resistance observed in response to olaparib and carboplatin/paclitaxel. Collectively, these data provide evidence to suggest that disrupting CSC’s ability to rely on mitochondria as an energy source may augment the effect of current treatment strategies.

We and others have shown that treatment with carboplatin and paclitaxel results in an enrichment of CSCs in a mouse model of OvCa as well as in patients undergoing treatment for HGSOC [63,64]. More recently, it was determined that treatment with PARP inhibitors could have a similar effect [50]. Specifically, treatment with olaparib or rucaparib preferentially induced cell death in the CD117− or CD133− fractions resulting in an enriched CD117+ or CD133+ fraction. Collectively, these data reinforced the need for combination treatment strategies that may augment carboplatin and paclitaxel or PARP inhibitors.

It is well recognized that under normal conditions, cells acquire energy for proliferation and/or survival through the absorption of nutrients broken down by glycolysis and oxidative phosphorylation [26,31,38]. Normal cells rely on oxidative phosphorylation as the major energy provider for cells since it yields more ATP molecules than glycolysis. In contrast, cancer cells, which frequently reside and proliferate in hypoxic conditions, often rely more on glycolysis [31,32]. Even in the presence of oxygen, there is evidence that cancer cells will preferentially use glycolysis. Known as the Warburg effect, the use of anaerobic metabolism, even though it is less efficient than oxidative phosphorylation in terms of ATP production, appears to benefit high proliferating cells and their metastatic properties by producing elevated lactate levels that acidify the tumor microenvironment [31,65]. CSCs, however, appear to defy this concept. There is growing endorsement for the idea that mitochondrial oxidative phosphorylation is their preferred metabolic method. This was initially demonstrated when investigators compared metabolic differences between glioma cancer stem and non-stem cells [66,67]. The glioma CSCs preferentially relied on oxidative phosphorylation. In contrast, the non-stem glioma tumor cells relied on aerobic glycolysis. Moreover, when oxidative phosphorylation was inhibited, they switched to glycolysis, confirming the CSC ability to adapt to different energy sources. This concept is not without controversy, however, and there are studies supporting and contrasting this theory [35,36,66,68]. Some of the contrasting data likely can be attributed to model differences (i.e., primary versus established cell lines). Recently, it was suggested that ovarian CSCs preferentially use oxidative phosphorylation and resist glucose deprivation [49]. CPI-613, also known as devimistat, is a lipase derivative that inhibits mitochondrial enzymes pyruvate dehydrogenase and alpha-ketoglutarate dehydrogenase, enzymes involved in mitochondrial metabolism. If ovarian CSCs are inherently more reliant on the mitochondrial oxidative phosphorylation or readily adapt to it under stress, then disruption of this pathway might serve to deplete the cells’ energy promoting cell death or at a minimum rendering them more susceptible to death. In our investigation, treatment of HGSOC cells with platinum and paclitaxel resulted in a decrease in the percent of live cells in the bulk tumor cell population, but flow cytometric analysis revealed an enrichment of CSCs in the remaining fraction. This increase was similar to what we had seen previously with no increase in cells undergoing mitosis, but the CSC fractions displayed an extended mitotic and Gap2 (M/G2) phase [50]. It is presumed that this delay allows the activation of a more efficient DNA repair machinery and their survival to PARP inhibitor treatment. Collectively these findings supported the concept that CSCs can adapt as needed to overcome drug selection pressure in their microenvironment. CSC’s ability to utilize or adapt to different energy sources is an example of just one of their survival mechanisms. Utilizing in vitro and in vivo models, we demonstrated that CPI-613 preferentially targets the CSC population. Using the CD133+ and CD117+ cell enrichment effect of the sphere-forming culture conditions for which carboplatin/paclitaxel had little effect as a positive control, we showed that CPI-613 treatment in vitro for 7 days induced a decrease in CD133+ and CD117+ cell frequency. Moreover, cell viability analysis of the different cell fractions discriminating for CD133/CD117 markers confirmed the CPI-613 target effect on CSCs since it did not induce any significant amount of cell death in the CD133−, CD117− cells. Appreciating the controversy surrounding the use of CSC markers as sole proof of a stem-like phenotype and the lack of universal CSC markers, we analyzed the CPI-613 effect on the more accepted cancer stem-like properties, such as sphere-forming ability and increased tumorigenicity rate in vivo. Cells able to survive the CPI-613 treatment showed a decrease in sphere-forming capacity in low adhesion plates and in the absence of serum. Furthermore, CPI-613 treatment decreases CD133+ and CD117+ cell frequency in the CSC-rich spheres compared to the lack of effect of vehicle and carboplatin/paclitaxel treatment. More interesting was the finding that pretreatment of the cells in a monolayer culture with CPI-613 before injection into an immunocompromised mouse model resulted in a decrease in tumorigenicity compared to vehicle pretreated cells. These results support the proposed beneficial effect of a metabolic inhibitor on targeting CSCs that promote tumor initiation.

Combination drug therapy has improved the probability and magnitude of therapeutic responses with the hope of reducing the likelihood of acquired resistance. Given that a metabolic inhibitor that disrupts oxidative phosphorylation might preferentially negatively affect CSCs, we wanted to test whether or not combining CPI-613 with either carboplatin/paclitaxel or a PARP inhibitor would provide some benefit over treatment with either carboplatin/paclitaxel or PARP inhibition alone. Single-agent CPI-613 was able to blunt tumor growth. However, what was more important was the finding that combining the metabolic inhibitor with the cytotoxics or the PARP inhibition negatively impacted the CSC populations, which were normally resistant to carboplatin/paclitaxel and/or enhanced by olaparib.

## 4. Materials and Methods

### 4.1. Cell Lines and In Vitro Culture

Human OvCa cell lines UWB1.289 MUT (*BRCA1* mutant, 2594delC germline mutation in exon 11 and deletion of the wild type allele; RRID: CVCL_B079), UWB1.289 WT (RRID: CVCL_B078) were purchased from the American Type Culture Collection (ATCC; Manassas, VA, USA). Human OvCa cell line PEO1 (BRCA2 mutated, homozygous mutation 5193C>G; RRID: CVCL_2686) was purchased from Sigma–Aldrich (St. Louis, MO, USA). OVCAR4 (RRID: CVCL_1672) and OVCAR3 (RRID: CVCL_0465) were provided by the National Cancer Institute – Developmental Therapeutics Program (NCI-DTP; Rockville, MD, USA). All cell lines were routinely tested for *Mycoplasma,* cultivated at 37 °C in 5% CO_2_ humidity, and passaged until passage 12. UWB1.289 MUT and UWB1.289 WT were maintained in 50% RPMI 1640 (GIBCO, Life Technologies; Carlsbad, CA, USA), 50% MEGM (Mammary Epithelial Growth Medium, MEGM Bullet Kit CC-3150; Lonza, Walkersville, MD, US), 10% FBS (GIBCO, Life Technologies; Carlsbad, CA, USA), and 1% Pen/Strep (Thermo Fisher Scientific; Waltham, MA, USA). UWB1.289 WT is a stable cell line derived from UWB1.289 MUT (ATCC CRL-2945), in which BRCA1 function was restored through transfection with a plasmid carrying the wild-type *BRCA1* gene; selection was maintained by culturing the cells in 200 µg/mL of G-418 (Life Technologies; Carlsbad, CA, USA). OVCAR3 cells were maintained in RPMI 1640 (GIBCO), 10% FBS (GIBCO), 1% Pen/Strep (Thermo Fisher Scientific), and 0.01 mg/mL of bovine insulin (Sigma–Aldrich). OVCAR4 cells were maintained in RPMI 1640 (GIBCO), 10% fetal bovine serum (FBS) (GIBCO), and 1% Pen/Strep (Thermo Fisher Scientific). PEO1 cells were maintained in RPMI 1640 (GIBCO), 10% FBS (GIBCO), 1% Pen/Strep (Thermo Fisher Scientific), and 2 mM Sodium Pyruvate (GIBCO).

### 4.2. Drug Treatment (Carboplatin, Paclitaxel, Olaparib, and CPI-613)-MTT and Cell Counting

Olaparib and CPI-613 were purchased from Selleckchem (Houston, TX, USA), and carboplatin and paclitaxel were obtained from Sigma (St. Louis, MO, USA). Olaparib and carboplatin concentrations were as previously described [50]. IC_50_ value (the concentration required to kill and/or inhibit proliferation of cells by 50% as compared to untreated control wells) of paclitaxel and CPI-613 drug was determined by 3-(4,5-dimethylthylthiazol2-yl)-2,5-diphenyltetrazolium bromide (MTT) analysis. All the cell lines were plated in 96-well plates (10,000 cells/well in 150 µML media). After overnight incubation, paclitaxel (0.5 nM, 1 nM, 2 nM, and 5 nM) or CPI-613 (50 µM, 75 µM, and 100 µM) were as single agents to each well. Six replicates were performed for each cell line. Cell viability was measured after 7 days using the MTT assay. A calibration curve was prepared using the data obtained from wells that contained a known number of cells. Cell proliferation was assessed by trypan blue (Thermo Fisher Scientific) staining and cell counting following treatment with the indicated concentrations of paclitaxel and CPI-613. Paclitaxel treatment was once per week, and CPI-613 treatment was over 7 d with replenishment of drugs every 2 d due to the half-life of the drug.

### 4.3. Flow Cytometry (CD133, CD117, Cell Viability)

Cells were stained with Live-Dead (Pacific Blue, 1:600; Invitrogen, Carlsbad, CA, USA) to discriminate living cells. Apoptotic and necrotic cells were discriminated using the Annexin/PI staining kit (Roche; Basel, Switzerland) followed by flow cytometry. The following anti-human monoclonal antibodies were used to discriminate CD133+ and CD117+ cells: anti-CD133 (CD133/2 clone 293C3, 1:10, PE-conjugated; Miltenyi Biotec, Auburn, CA, USA; RRID: AB_2661207) and anti-CD117 (clone A3C6E2, 1:10, APC-conjugated; Miltenyi Biotec; RRID: AB_2660103). Following incubation with FcR blocking reagent (Miltenyi Biotec), cells were resuspended in PBS buffer (PBS, 2% FBS and 1 mM EDTA) and stained with the relevant antibodies. Respective IgG isotype antibodies were included as negative controls in the first analysis and antibody titration analyses. In subsequent experiments, unstained cells were included as a control for background fluorescence. Cells were analyzed using a using FACS LSRII cytofluorimeter (BD Biosciences, Franklin Lakes, NJ, USA). Data were collected from at least 1 × 10^5^ live cells/sample and analyzed with FlowJo 10.1 version (TreeStar, Ashland, OR, USA).

### 4.4. Sphere Formation and ELDA

The cells were cultured in DMEM-F12 (GIBCO) medium without serum at a density of 2 × 10^5^ cells/well in ultra-low-attachment six-well plates (Corning, Inc., Corning, NY, USA). Human recombinant epidermal growth factor (EGF; 10 ng/mL) and basic fibroblast growth factor (bFGF; 10 ng/mL) were replenished every 3 d. CD133 and CD117 positive and negative cells were determined by flow-cytometry after 14 d of culture under these conditions and compared to cells maintained in a monolayer culture in the presence of serum. Photomicrographs of spheroids were captured with the microscope Nikon Eclipse TE2000 at different magnifications. After 14 d of sphere formation, the spheroids derived from UWB1.289 MUT and OVCAR3 cell lines were treated over a period of 7 d with 75 µM CPI-613 every 3 d and with different concentrations of carboplatin/paclitaxel or their respective vehicles. Again, CD133 and CD117 positive and negative cells were determined by flow-cytometry. The extreme limiting dilution assay (ELDA) was performed to analyze the sphere-forming efficiency after CPI-613 treatment in vitro. Cells were treated under monolayer culture conditions with CPI-613 (75 µM), harvested after 7 d, and plated in ultra-low-attachment 96-wells plates (Corning, Inc.) at different cell densities (1, 10, 50, 100 cells per well × 24 wells of each:). Cells were maintained for 10 d in DMEM-F12 without serum, and bFGF and EGF growth factors were added to the culture medium every 3 d. The number of spheres was counted 10 d after the establishment of the cultures, and statistical analysis was performed using the ELDA software (http://bioinf.wehi.edu.au/software/elda/).

### 4.5. Tumorigenicity In Vivo Assay

To analyze the in vivo tumorigenicity rate after CPI-613 pretreatment in vitro, OVCAR3 cells were treated in vitro with either CPI-613 (75 µM) or vehicle every 72 h. Cells were harvested after 7 d and 1 × 10^6^ cells were injected respectively in 5 mice for each arm: vehicle and CPI-613 pretreated. The tumorigenicity rate was analyzed after 21, 35 and 48 d. All mice were euthanized with CO_2_ after 48 d.

### 4.6. In Vivo Experiment 

Using an institutionally approved Institutional Care and Use Committee (IACUC) protocol (2017N0000236), twelve-week old NOD/SCID mice were subcutaneously injected with 3 × 10^6^ OVCAR3 cells 1:1L PBS:Matrigel (Corning Matrigel, BD Biosciences). Measurements of the resulting tumors were determined by calipers every other day, and the bodyweight of each mouse was assessed twice per week. The tumor volume was calculated using the following formula: (width^2^ × height)/2. When the tumor volume reached 150 to 200 mm^3^, the mice were randomly divided into four arms. The treatments included vehicle, carboplatin/paclitaxel (25 mg/kg and 7 mg/kg, respectively), and CPI-613 (12.5 mg/kg) as single agents or carboplatin/paclitaxel in combination with CPI-613. A second in vivo 4 arm experiment was conducted only the treatments included vehicle, olaparib (50 mg/kg), and CPI-613 (25 mg/kg) as single agents or olaparib in combination with CPI-613 (25 mg/kg). Both the experiments were 14 days in length, and treatments were administered via intraperitoneal injection (carboplatin/paclitaxel and CPI-613 weekly administration, olaparib daily administration).

Tumor volume was measured every three days. At the completion of the experiment, mice were euthanized in accordance the with IACUC approved protocol, and xenografts were harvested. Portions of each xenograft were snap-frozen as well as formaldehyde-fixed and paraffin-embedded for further analyses. Tumors were processed following a previously described protocol [69] (and H-2K^d+^ mouse cells were removed using a fluorescein isothiocyanate (FITC) conjugated antibody (BD Biosciences) and Macs LD columns (Miltenyi Biotec) as per manufacturers’ recommendations. H-2K^d-^ cells were stained with Live-Dead (Pacific Blue, 1:600; Invitrogen), anti-CD133 (CD133/2 clone 293C3, 1:10, PE-conjugated; Miltenyi Biotec) and anti-CD117 (clone A3C6E2, 1:10, APC-conjugated; Miltenyi Biotec) and analyzed using FACS LSRII cytofluorimeter (BD Biosciences). Data were collected from at least 1 × 10^5^ live cells/sample and analyzed with FlowJo 10.1 version (TreeStar).

### 4.7. Western Blotting

Whole-cell lysates were prepared from cell lines or dry frozen xenograft tissue samples with mammalian protein extraction reagent (Thermo Scientific, Tewksbury, MA, USA) lysis buffer supplemented with phosphatase, protease, and kinase inhibitors (all from Sigma–Aldrich, St. Louis, MO, USA). The protein concentration of the resulting supernatants was assessed, and two to five micrograms of protein lysates were resolved on 10%, Bis-Tris gels (NuPAGE Novex, Life Technologies; Carlsbad, CA, USA) and transferred to polyvinylidene difluoride (PVDF) membranes (Millipore, Billerica, MA, USA). Following transference, assessment of protein loading was visualized using Ponceau S solution (Sigma–Aldrich, St. Louis, MO, USA), and membranes were then blocked in 5% non-fat milk in TBST. Primary antibodies directed against at Phospho-AMPKα (Thr172) (40H9) at a 1:1000 dilution (2535, Cell Signaling, Danvers, MA, USA), Anti-Pyruvate Dehydrogenase E1-alpha subunit (phospho S293) (EPR12200) at a 1:1000 dilution (ab177461), and Anti-Pyruvate Dehydrogenase E1-alpha subunit (9H9AF5) at a 1:2000 dilution (abc110330, both from Abcam, Cambridge, MA, USA) were incubated overnight at 4 °C. Equal protein loading was assessed using either GAPDH (5174) or β-tubulin (2128) at a 1:5000 dilution (Cell Signaling). Membranes were then incubated with either a mouse or rabbit horseradish peroxidase (HRP)-conjugated secondary antibody at a 1:10,000 (Santa Cruz Biotechnology, Dallas, TX, USA) and developed using a chemiluminescent detection reagent (ProSignal ™ Dura, Genesee Scientific, San Diego, CA, USA). All images were captured using the BioRad ChemiDoc ™ Imaging system and analyzed using Image J (Bethesda, MD, USA).

### 4.8. Statistics

Data were analyzed with GraphPad Prism 6.0 (GraphPad Software, La Jolla, CA, USA). Data from replicate experiments are shown as mean values ± standard error. Comparisons between groups were analyzed by a two-tailed Student’s *t*-test or two-way ANOVA, as appropriate. A *p*-value < 0.05 was considered statistically significant.

## 5. Conclusions

Collectively, our results reveal that CD133+ and CD117+ OvCa CSCs can be effectively targeted using a CPI-613 drug through the inhibition of their peculiar mitochondrial metabolism. We suggest a potential complementary effect of pairing a metabolic inhibitor with classical cytotoxic drugs or a PARP inhibitor to overcome the unintended CSC enrichment induced by these therapeutic strategies. These data provide a preclinical rationale to develop and clinically test treatment strategies that pair a metabolic inhibitor with a conventional cytotoxic or PARP inhibitor. This combination holds the promise of reducing bulk cells while simultaneously targeting the CSC population that may serve to replenish daughter cells preventing tumor resurgence and leading to more durable responses.

## Supplementary Materials

The following are available online at https://www.mdpi.com/2072-6694/11/11/1678/s1, Figure S1: CD133+ and CD117+ cells from ovarian cancer cell lines demonstrate a phenotypic and functional profile compatible with CSCs, Figure S2: Annexin/PI analysis from the in vivo experiment of CPI-613 and carboplatin/paclitaxel combination, Figure S3: Western Blot Analysis of UWB1.289 MUT cells treated with 75 µM CPI-613, Table S1: Relevant properties of the analyzed OvCa cell lines are shown including the original tumor histology, platinum response, *TP53* and *BRCA* gene status and frequency of cells expressing stemness markers CD133 and CD117.

## Abbreviations

OvCa	ovarian cancer
EOC	epithelial ovarian cancer
CSCs	cancer stem cells
OXPHOS	oxidative phosphorylation
TCA	tricarboxylic acid
PDH	pyruvate dehydrogenase
KGDH	alpha-ketoglutarate dehydrogenase
FDA	Food and Drug Administration
AML	acute myeloid leukemia
MDS	myelodysplastic syndromes
PTCL	peripheral T-cell lymphoma
ROS	reactive oxygen species
PARPi	PARP inhibitors
ATP	PARP inhibitors
