# Supplementary material for: The Metabolic Inhibitor CPI-613 Negates Treatment Enrichment of Ovarian Cancer Stem Cells
Source: Cancers (Basel). 2019 Oct 29;11(11):1678. doi: 10.3390/cancers11111678 (PMC6896080; doi:10.3390/cancers11111678)
Supplement: Supplementary file 1 [file cancers-11-01678-s001.pdf]

Supplementary Materials: The Metabolic Inhibitor, CPI-613, Negates Treatment Enrichment of Ovarian Cancer Stem Cells

Chiara Bellio, Celeste DiGloria, David R. Spriggs, Rosemary Foster, Whitfield B. Growdon and Bo R. Rueda

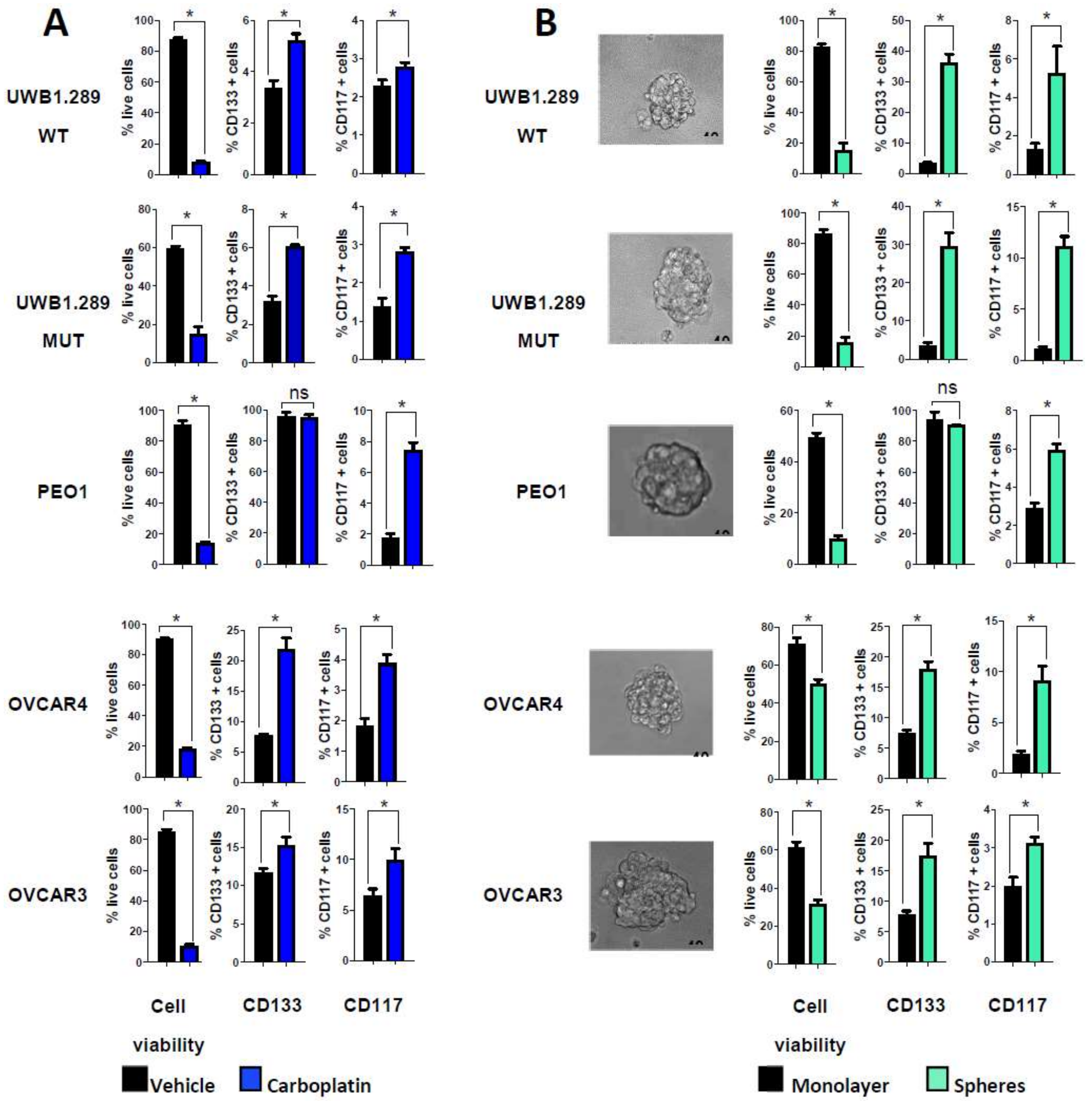

Figure S1. CD133+ and CD117+ cells from ovarian cancer cell lines demonstrate a phenotypic and functional profile compatible with CSCs. (**A**) Percentage of viable (left panels), CD133+ (center panels) and CD117+ (right panels) cells in the indicated cell lines 72 h following incubation with either 5 μM (UWB1.289 MUT and UWB1.289 WT) or 10 μM (PEO1, OVCAR4 and OVCAR3) of carboplatin as determined by flow cytometry. * *p*-value < 0.05; (**B**) Flow cytometric determination of the frequency of viable (left panels), $CD133^+$ (center panels) and $CD117^+$ (right panels) cells in the indicated cell lines following culturing under monolayer or sphere forming conditions as described in Materials and Methods. The graphs show the mean percentage of the indicated cell population ± SEM. For each cell line, a representative image of a sphere that formed under low adhesion/no serum culture conditions is included. * *p*-value < 0.05.

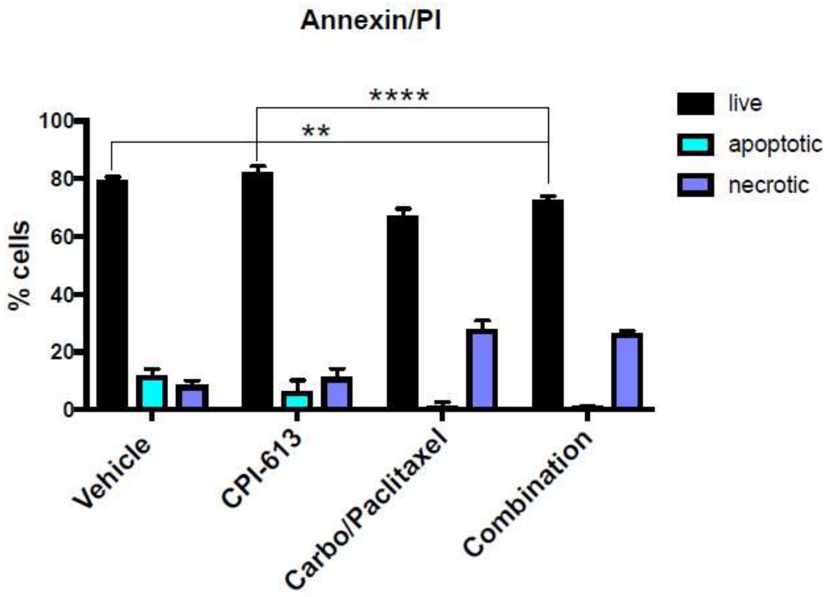

Figure S2. Annexin/PI analysis from the in vivo experiment of CPI-613 and carboplatin/paclitaxel combination. Tumors were harvested at the end of treatment period and cells were stained with Annexin/PI (middle panel) to assess cell viability. ** *p*-value < 0.01; **** *p*-value < 0.0001.

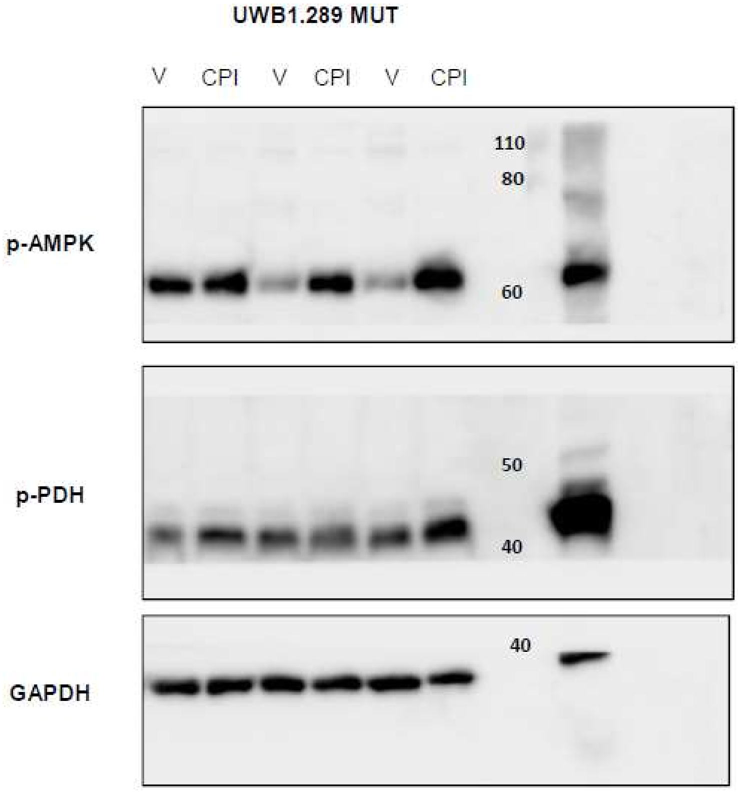

p-AMPK

Vehicle	CPI-613	
**1,10102733**	**1,11393854**	**set 1**
**0,9723443**	**0,98766411**	
**1,35229425**	**1,15924394**	
0,56864889	0,80371024	set 2
1,03731752	1,05956621	
0,7160007	0,86213672	
0,90441967	0,87141958	set 3
0,82199514	0,89745683	
0,86380664	1,09931205	

p-PDH

Vehicle	CPI-613	
**0,51398093**	**0,75053834**	**set 1**
**0,61300126**	**0,69358566**	
**1,02478311**	**1,09563186**	
1,0062845	0,91133304	set 2
0,97047547	0,98868437	
0,68911872	0,77411497	
0,68752698	0,8511386	set 3
0,89524834	0,98179745	
0,79675007	0,87920779	

Figure S3. Western Blot Analysis of UWB1.289 MUT cells treated with 75 μM CPI-613. Assessment of lysates collected 2 h post treatment revealed a significant increase in the expression of p-PDH and p-AMPK compared to vehicle. We used the same blot for multiple westerns. After transferring we used Ponceau Red Stain to visualize protein loading, confirm transfer, and ladder marker in correlation with protein bands. We used a ruler and cut the blot based on which proteins were of interest. We used the 293T cell line as positive control. We ran three different sets of samples from UWB1.289 MUT cell line. The levels were determined from the densitometry readings and graphed. Values were normalized on the respective densitometry reading of GAPDH.

Table S1. Relevant properties of the analyzed OvCa cell lines are shown including the original tumor histology, platinum response, *TP53* and *BRCA* gene status and frequency of cells expressing stemness markers CD133 and CD117.

Cells	Histology	Platinum Response	*TP53* Gene Status	*BRCA* Gene Status	Frequency Cells Expressing CD133	Frequency Cells Expressing CD117
UWB1.289 MUT	Serous	Sensitive	Mutated	*BRCA1* mutated	3.18 ± 1.5	1.06 ± 0.5
UWB1.289 WT	Serous	Sensitive	Mutated	NO mutation	3.33 ± 1	2.57 ± 0.5
PEO1	Serous	Sensitive	Mutated	*BRCA2* mutated	95.7 ± 2	1.2 ± 1.5
OVCAR4	Serous	Sensitive	Mutated	NO mutation	7.16 ± 0.5	1.8 ± 0.5
OVCAR3	Serous	Sensitive	Mutated	NO mutation	12.8 ± 1.5	6.43 ± 1.5

© 2019 by the authors. Licensee MDPI, Basel, Switzerland. This article is an open access article distributed under the terms and conditions of the Creative Commons Attribution (CC BY) license (http://creativecommons.org/licenses/by/4.0/).
